# Systemic treatment with CAR-engineered T cells against PSCA delays subcutaneous tumor growth and prolongs survival of mice

**DOI:** 10.1186/1471-2407-14-30

**Published:** 2014-01-18

**Authors:** Victoria Hillerdal, Mohanraj Ramachandran, Justyna Leja, Magnus Essand

**Affiliations:** 1 Department of Immunology, Genetics and Pathology, Rudbeck Laboratory, Uppsala University SE-75185 Uppsala, Sweden

**Keywords:** CAR T cells, PSCA, Genetic engineering, Prostate cancer, Adoptive transfer

## Abstract

**Background:**

Adoptive transfer of T cells genetically engineered with a chimeric antigen receptor (CAR) has successfully been used to treat both chronic and acute lymphocytic leukemia as well as other hematological cancers. Experimental therapy with CAR-engineered T cells has also shown promising results on solid tumors. The prostate stem cell antigen (PSCA) is a protein expressed on the surface of prostate epithelial cells as well as in primary and metastatic prostate cancer cells and therefore a promising target for immunotherapy of prostate cancer.

**Methods:**

We developed a third-generation CAR against PSCA including the CD28, OX-40 and CD3 ζ signaling domains. T cells were transduced with a lentivirus encoding the PSCA-CAR and evaluated for cytokine production (paired Student’s t-test), proliferation (paired Student’s t-test), CD107a expression (paired Student’s t-test) and target cell killing *in vitro* and tumor growth and survival *in vivo* (Log-rank test comparing Kaplan-Meier survival curves).

**Results:**

PSCA-CAR T cells exhibit specific interferon (IFN)-γ and interleukin (IL)-2 secretion and specific proliferation in response to PSCA-expressing target cells. Furthermore, the PSCA-CAR-engineered T cells efficiently kill PSCA-expressing tumor cells *in vitro* and systemic treatment with PSCA-CAR-engineered T cells significantly delays subcutaneous tumor growth and prolongs survival of mice.

**Conclusions:**

Our data confirms that PSCA-CAR T cells may be developed for treatment of prostate cancer.

## Background

Adoptive transfer of *ex vivo*-expanded tumor infiltrating lymphocytes (TILs) has shown promising results as a treatment of human cancers [1]. However, since it is not possible to isolate and expand TILs from all patients and tumor types, an attractive alternative technology is to isolate T cells from peripheral blood of a cancer patient and genetically engineer them with a novel T cell receptor (TCR), recognizing a tumor-associated antigen in the context of human leukocyte antigen (HLA) presentation, or a chimeric antigen receptor (CAR), recognizing a tumor-associated antigen on the surface of tumor cells. The engineered T cells are expanded and adoptively transferred back to the patient. Engineered T cells with specificities for a variety of tumor-associated antigens have been developed [2]. The first successful attempt to treat cancer patients with TCR-engineered T cells was reported from the Surgery Branch at the National Cancer Institute in 2006, where 2 patients out of 15 (13%) demonstrated objective regression of metastatic melanoma lesions when treated with MART-1-TCR-engineered autologous T cells [3]. CARs are artificial single chain antibody fragment (ScFv)-based receptors linked to a signaling domain for T cell activation [4]. First-generation CARs contain the CD3 ζ chain signaling domain from the TCR complex for T cell activation, whereas second-generation CARs include also a second co-stimulatory signaling domain from CD28 [5], 4-1BB [6], OX-40 [7] or CD27 [8]. Third-generation CARs contain two co-stimulatory signaling domains along with the CD3 ζ chain [9]. A successful report with complete remission of two out of three B-cell chronic lymphocytic leukemia (CLL) patients using CD19-CAR T cells was reported from University of Pennsylvania in 2011 [10]. This was followed up by successful treatment also of B-cell acute lymphocytic leukemia (ALL) in 2013 [11].

Prostate cancer is one of the most common cancers in the developed world. Curative treatment is not possible when the tumor has spread beyond the prostate gland. Since the prostate is a dispensable organ, T cell immunotherapy is an attractive approach for treatment of prostate cancer as it allows for targeting of tissue-specific antigens that are also expressed on malignant prostatic cells. Prostate stem cell antigen (PSCA) is a prostate tissue-restricted antigen highly expressed on primary and metastatic prostate cancer cells [12]. PSCA has been evaluated as a DNA vaccine in an experimental model for prostate cancer [13] and T cell epitopes from PSCA have been identified [14]. Furthermore, HLA-A2-positive prostate cancer patients have been found to have circulating T cells against PSCA [15]. Positive results have been reported in a study using a bi-specific antibody against PSCA and CD3, thereby re-directing T cells towards PSCA-expressing cells [16]. Humanized anti-PSCA antibodies have entered clinical trials [17,18]. Herein, we use a third-generation CAR targeting PSCA, which besides the CD3 ζ chain contains the signaling domains of CD28 and OX-40. We evaluate whether primary T cells from peripheral blood of healthy volunteers transduced with a lentiviral vector encoding the PSCA-CAR molecule are able to recognize and kill cancer cells expressing PSCA both *in vitro* and *in vivo*.

## Methods

### Lentivirus vector design and lentivirus production

Lentivirus for target cell modification: A number of third-generation self-inactivating lentiviral plasmids expressing two transgenes separated by the sequence for the *Thosea asigna* virus 2A (T2A) peptide were constructed using pGreenPuro (SBI System Biosciences, Mountain View, CA). The plasmids are denoted pBMN(TurboRFP-Luc2), pBMN(copGFP-PSCA) and pBMN(copGFP-TARP), where TurboRFP encodes turbo red fluorescent protein, Luc2 encodes codon-optimized *firefly* luciferase, copGFP encodes *copepod* green fluorescent protein, PSCA encodes the human prostate stem cell antigen and TARP encodes human T cell receptor γ-chain alternate reading frame protein.

Lentivirus for T cell engineering: An anti-PSCA CAR-expressing lentiviral plasmid, pBMN(PSCA-CAR), was generated by fusing a PSCA-recognizing single chain antibody fragment, obtained through reversed genetics [19] with the signaling moieties of CD28, OX-40 and CD3 ζ chain, from a plasmid obtained from M Brenner, Baylor College of Medicine, Houston, TX [20].

Lentiviruses were produced in HEK-293 T cells using polyethyleneimine (Sigma-Aldrich, St Louis, MO) transfection. The pBMN-based lentiviral plasmid and the packaging plasmids pLP1, pLP2 and pVSV-G (Invitrogen) were used at a ratio of 2:1:1:1. The supernatant was harvested 48 and 72 hours post-transfection, concentrated through ultracentrifugation at 75,000 × *g* for 90 minutes and stored at -80°C. Mock lentivirus was produced using an empty pRRL lentiviral plasmid (Addgene, Cambridge, MA).

### Target cell lines

The mel526 cell line was obtained from T Boon, Ludwig Institute for Cancer Research, Brussels, Belgium and cultured in Dulbecco's Modified Eagle Medium (DMEM) supplemented with 10% fetal bovine serum (FBS) (Invitrogen, Carlsbad, CA). Mel526-based target cells were produced through lentiviral transduction followed by sorting using a FACS Aria III sorter (BD Biosciences, Franklin Lakes, NJ). Mel526 cells co-expressing TARP, copGFP, Luc2 and turboRFP will be referred to in the text as mel526(TARP), and mel526 cells co-expressing PSCA, copGFP, Luc2 and turboRFP will be referred to as mel526(PSCA).

### T cells from activated and lentivirus transducted of PBMCs

Peripheral blood mononuclear cells (PBMCs) were isolated from buffy coats from healthy donors using Ficoll-Paque (GE Healthcare, Uppsala, Sweden) and cultured in RPMI-1640 supplemented with 10% human AB serum (our own production), 2 mM L-glutamine, 10 mM HEPES, 20 μM β-mercaptoethanol and 1% penicillin/streptomycin. The PBMCs were activated with 100 ng/ml OKT-3 (Nordic Biosite, Täby, Sweden) and 100 IU/ml IL-2 (Proleukin, Novartis, Basel, Switzerland) for 2 days to selectively stimulate T cells. Activated cells were transduced with 50 μl concentrated PSCA-CAR-encoding lentivirus or Mock lentivirus for 4 hours at 37°C in the presence of 10 μg/ml protamine sulphate and 100 IU IL-2 (Sigma-Aldrich). Transduction was repeated 24 hours later and the cells were cultured and expanded for 2-4 weeks before analysis. For analysis of PSCA-CAR expression, cells were stained with biotinylated protein-L (Genscript, Piscataway, NJ) [21], washed 3 times with PBS containing 4% BSA, followed by labeling with phycoerythrin (PE)-conjugated streptavidin (BD Biosciences) or stained with Alexa Fluor® 647 F(ab')2 Fragment of Goat Anti-Mouse IgG (H + L) (Invitrogen) and stained with an allophycocyanin (APC)-conjugated anti-CD3 or fluorescein isothiocyanate (FITC)-conjugated anti-CD3 antibody (Nordic Biosite). Flow cytometry analysis was performed using FACSCanto II or LSRII (BD Biosciences).

### IFN-γ and IL-2 ELISA

Activated and PSCA-CAR-transduced or Mock-transduced T cells (10^5^ cells) were co-cultured with mel526(PSCA) or mel526(TARP) cells at a 1:1 ratio in 96-well plates. Supernatants were collected after 16 hours. ELISA (Mabtech, Nacka Strand, Sweden) was used to detect IFN-γ and IL-2 secretion.

### Proliferation assay

Activated and PSCA-CAR-transduced or Mock-transduced T cells (10^5^ cells) were labeled for 20 minutes at 37°C with 5 μM Cell Trace Violet (Invitrogen) in PBS and then washed with cold cell culture medium containing 10% human serum to stop the labeling reaction. Labeled PBMCs were co-cultured with irradiated (50 Gy) mel526(PSCA) or mel526(TARP) cells at a 1:1 ratio in 96-well plates for 5 days. The T cells received a low dose of IL-2 (10 IU/ml) on day 1. The labeled cells were then collected and stained with an APC-conjugated anti-CD3 antibody followed by flow cytometry analysis.

### CD107a degranulation flow cytometry analysis

Activated and PSCA-CAR-transduced or Mock-transduced T cells (10^5^ cells) were co-cultured with mel526(PSCA) or mel526(TARP) cells at a 1:1 ratio in 96-well plates for 16 hours. Cells were stained with a FITC-conjugated anti-CD107a antibody and an APC-conjugated anti-CD3 antibody followed by flow cytometry analysis.

### Bioluminescence *in vitro* killing assay

Activated and PSCA-CAR-transduced or Mock-transduced T cells were co-cultured with luciferase-expressing mel526(PSCA) or mel526(TARP) (15000 cells) in various effector to target cell ratios (0.4:1–50:1) in flat-bottomed 96-well plates. Co-cultures were harvested 48 hours later and analyzed for luciferase expression using Steady-Glo® Luciferase Assay System (Promega Corporation, Madison, WI), according to the manufacturer’s instruction, and the luminescence was measured in a luminometer (Wallac Victor 2 Multi-label Counter, Perkin Elmer, Waltham, MA). Luciferase activity from target cells not exposed to T cells was set as 100% cell viability (survival).

### Animal model for T cell treatment

Nude NMRI mice (Harlan, Netherlands) were injected subcutaneously (hind flank) with 3 × 10^6^ mel526(PSCA) cells. One, seven and fourteen days later the mice received intravenous injection of 1 × 10^7^ PSCA-CAR-transduced T cells or Mock-transduced T cells. Twelve mice per group were used. The tumors were measured by caliper and tumor volume was calculated using the equation (length × width^2^)/2. Animals were sacrificed, when tumors reached over 1000 mm^3^. The Uppsala Animal Ethics Committee has approved the animal studies (ID numbers C319/9 and C195/11).

### Statistics

Statistics were performed using GraphPad prism software version 5.04 (La Jolla, CA, USA). Statistical analysis for IFN-γ and IL-2 secretion, cell proliferation and CD107a degranulation were performed using paired Student’s t-test. Log-rank test was used to compare survival curves created by the Kaplan-Meier method. Values of p < 0.05 were considered statistically significant.

## Results

### Transduced T cells efficiently express the PSCA-CAR molecule

**Figure 1:**
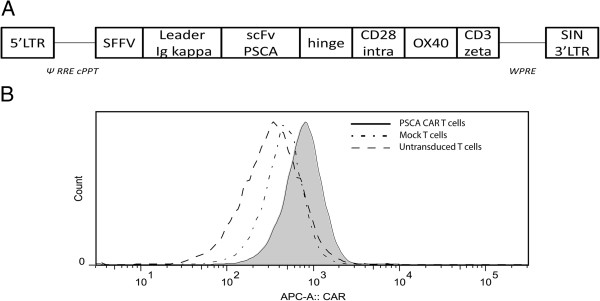
**Lentivirus cassette and surface expression of PSCA-CAR on T cells. (A)** The design of the PSCA-CAR-encoding lentiviral vector is shown. **(B)** Expression of the PSCA-CAR molecule on the surface CD3^+^ T cells after transduction with the lentiviral vector was analyzed by flow cytometry using Alexa-647 F(ab')2 Fragment of Goat Anti-Mouse IgG (H + L). The solid filled histogram represents PSCA-CAR expression of transduced T cells, complex histogram represents CAR expression on Mock lentivirus-transduced T cells and the dashed histogram represents untransduced control T cells.

The ScFv nucleotide sequence of the anti-PSCA molecule was synthesized by reverse genetics from the amino acid sequence presented in the reference by Reiter and co-workers [19] and cloned together with the rest of the CAR molecule into a pGreenPuro-derived lentiviral vector under transcriptional control of the spleen-focus forming virus (SFFV) promoter (Figure 1A). To improve signal transduction through the CAR molecule, the intracellular domains of CD28 and OX-40 were included [20] and fused to the CD3 ζ chain. The leader sequence from the immunoglobulin kappa light chain was included for efficient expression of the CAR molecule on the cell surface of transduced cells. The anti-PSCA ScFv sequence was separated from the signaling part of the CAR molecule with a hinge region from an IgG heavy chain to allow for better flexibility.

Peripheral blood lymphocytes isolated from healthy donors were activated for 24 hours and transduced with the PSCA-CAR-encoding lentivirus, or Mock lentivirus followed by two weeks of culture. The expression of PSCA-CAR was verified using Alexa-647 F(ab')2 Fragment of Goat Anti-Mouse IgG (H + L) (Invitrogen), which labels the heavy and light chain of mouse IgG and analyzed by flow cytometry analysis. T cells were efficiently transduced and expressed significant levels of PSCA-CAR when compared to Mock lentivirus-transduced or untransduced PBMCs (Figure 1B).

### PSCA-CAR T cells specifically secrete IFN-γ and IL-2 and proliferate when exposed to target cells expressing the PSCA antigen

We first wanted to evaluate the PSCA-CAR T cells, generated from peripheral blood, against target cells *in vitro*. We could not make use of prostate cancer target cell lines with endogenous PSCA as they have been reported to down-regulate expression of PSCA during *in vitro* culture [22]. We also screen a large number of prostate cancer cell line as well as primary prostate epithelial cells at different passages for PSCA expression by flow cytometry (Additional file 1: Methods) but were unable to detect any PSCA expression (Additional file 2: Figure S1A). Immunohistochemistry analysis has detected PSCA expression in pancreatic cancer cell lines [23]. However, we were unable to detect PSCA expression on the surface of pancreatic cancer cell lines by flow cytometry (Additional file 2: Figure S1B). There are reports that suggest that xenografted pancreatic cancer cell lines regain PSCA expression *in vivo*. We therefore, transplanted two human pancreatic cancer cell lines to NMRI nude mice, excised the grafts after 3–4 weeks, made single cell suspension and examined the cell-surface expression of PSCA by flow cytometry (Additional file 1: Methods). These cell lines did not regain PSCA cell surface expression (Additional file 3: Figure S2). Therefore, we took the approach to lentivirally transduce target cells (mel526) to express the relevant antigen, PSCA, or an irrelevant control antigen, TARP. Thereby we establish a target cell line with a stable and intermediate strong PSCA expression. PSCA expression levels on mel526(PSCA) is shown in Additional file 2: Figure S1C.

**Figure 2:**
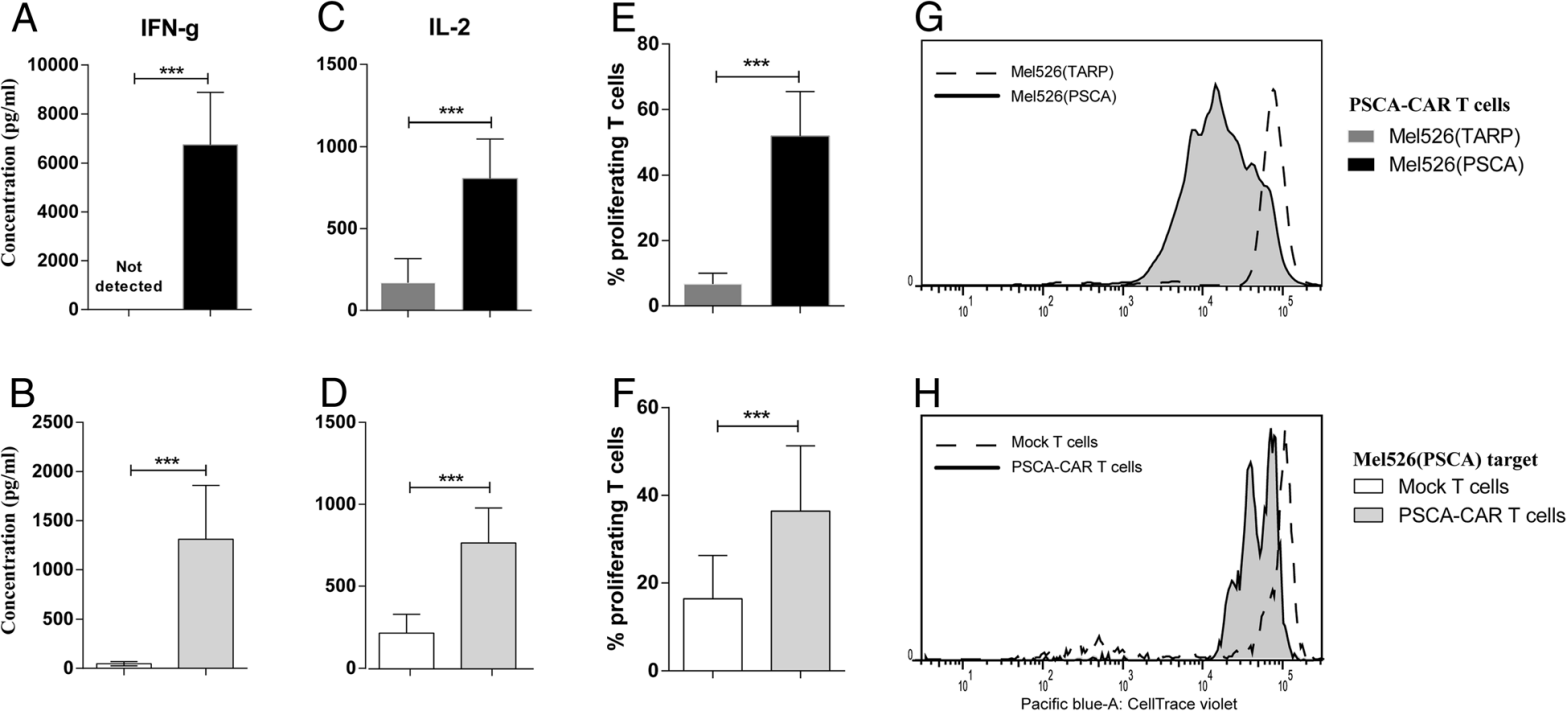
**Specific IFN-γ and IL-2 release and proliferation of PSCA-CAR-engineered T cells.** Lymphocytes were isolated from healthy donors and T cells were stimulated before being transduced with a lentiviral vector encoding the CAR against PSCA or Mock lentivirus. PSCA-CAR T cells were then co-cultured overnight with mel526(PSCA) or irrelevant mel526(TARP) target cells. In a separate experiment PSCA-CAR T cells and Mock T cells were co-cultured with relevant target mel526(PSCA) cells. ELISA was used to analyze IFN-γ release from **(A)** PSCA-CAR T cells against mel526(PSCA) or mel526(TARP) target cells (n = 4), **(B)** PSCA-CAR T cells and Mock T cells against mel526(PSCA) target cells (n = 4). ELISA was used to analyze IL-2 release from **(C)** PSCA-CAR T cells against mel526(PSCA) or mel526(TARP) target cells (n = 4), **(D)** PSCA-CAR T cells and Mock T cells against mel526(PSCA) target cells (n = 4). PSCA-CAR T cells and Mock T cells were labeled with a fluorescent dye and T cell proliferation, assessed as dilution of the dye for each cell division, was measured by flow cytometry after 5 days of co-culture with mel526(PSCA) target cells or mel526(TARP) control cells. Pooled T cell proliferation data for **(E)** PSCA-CAR T cells against mel526(PSCA) or mel526(TARP) target cells, **(F)** PSCA-CAR T cells and Mock T cells against mel526(PSCA) target cells (n = 4). **(G)** A representative histogram of proliferation for T cells established from one donor in the pool presented in **E**. **(H)** A representative histogram of proliferation for T cells established from one donor in the pool presented in **F**. Asterisks indicate significance (***p < 0.001, paired Student’s t-test). Error bars represent standard deviation from four individual donors run in triplicates.

PSCA-CAR T cells released high levels of IFN-γ (Figure 2A) and IL-2 (Figure 2C) in response to relevant mel526(PSCA) target cell exposure but not in response to the irrelevant mel526(TARP) target cells. In another control experiment it was observed that when PSCA-CAR T cells or Mock T cells were exposed to relevant target mel526(TARP) only PSCA-CAR T cells secreted IFN-γ (Figure 2B) and IL-2 (Figure 2D). We next evaluated the proliferative capacity of the PSCA-CAR T cells by labeling them with CellTrace Violet and exposing them to target cells. The PSCA-CAR T cells proliferated significantly better upon stimulation with mel526(PSCA) target cells than with mel526(TARP) cells, as detected through dilution of the dye and a lower intensity of the fluorescent signal. The results on proliferation from a pool of four donors are shown in Figure 2E and a representative image of proliferation for T cells established from one donor is presented in Figure 2G. The same proliferation pattern was observed when PSCA-CAR T cells were compared to Mock T cells co-cultured with relevant target mel526(PSCA) with pooled results in Figure 2F and a representative image in Figure 2H. The experiments with PSCA-CAR T cells and Mock T cells (Figures 2F, 2H) were performed with PBMCs isolated from different donors when compared to the experiments with relevant and irrelevant targets (Figure 2E,G).

### PSCA-CAR T cells specifically degranulate upon specific antigen recognition and kill PSCA-expressing target cells

**Figure 3:**
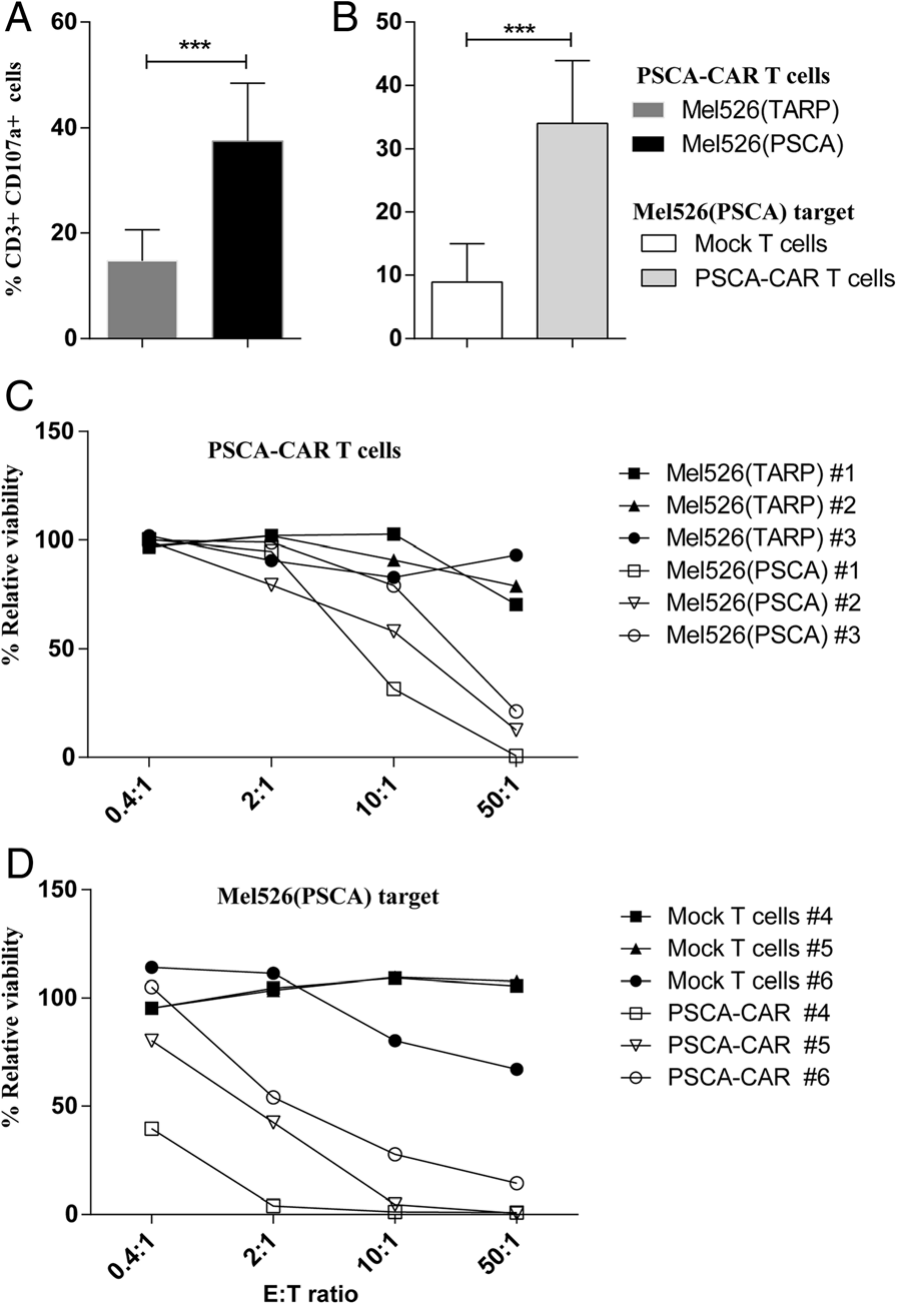
**PSCA-CAR-engineered T cells specifically degranulate upon antigen recognition and kill PSCA-expressing tumor cells.** PSCA-CAR T cells were co-cultured with mel526(PSCA) target cells or mel526(TARP) control cells. In a separate experiment PSCA-CAR T cells and Mock T cells were co-cultured with relevant target mel526(PSCA) cells. T cells (CD3^+^) were analyzed for CD107a expression (degranulation) by flow cytometry after 16 hours. Pooled data of CD107a expression on T cells from **(A)** PSCA-CAR T cells against mel526(PSCA) or mel526(TARP) target cells (n = 4), **(B)** PSCA-CAR T cells and Mock T cells against mel526(PSCA) target cells (n = 4). Asterisks indicate significance (*** p < 0.001, paired Student’s t-test). Error bars represent standard deviation from four individual donors run in triplicates. **(C)** PSCA-CAR T cells from three donors (#1, #2, #3) were co-cultured with luciferase-expressing mel526(PSCA) or mel526(TARP) target cells for 2 days. **(D)** PSCA-CAR T cells or Mock T cells from three donors (#4, #5, #6) were co-cultured with luciferase-expressing mel526(PSCA) for 4 days. Luciferase expression in target cells was then measured. Target cell viability was related to the luciferase signal for target cells not exposed to T cells. Error bars represent standard deviation from triplicate samples.

When an activated T cell recognizes its cognate antigen, it initiates killing of the target cell. During this process, the content of proteolytic granules are released from the cytoplasm of the T cell, creating pores within the cell membrane of target cells. The emptying of the granule vesicles in T cells is associated with translocation of molecules from the granules to the cell surface. We analyzed the cell surface expression of one such molecule, CD107a, which is also known as lysosomal-associated membrane protein (LAMP)-1. The PSCA-CAR-engineered T cells degranulated, as seen by CD107a surface expression, in response to mel526(PSCA) cells, but not in response to mel526(TARP) cells. When T cells from four donors were pooled we found that a significantly higher proportion of PSCA-CAR-engineered T cells degranulated when exposed to mel526(PSCA) target cells compared to mel526(TARP) cells (Figure 3A). We also observed that Mock T cells degranulated to a significantly lesser degree when exposed to relevant target mel526 (PSCA) when compared to PSCA-CAR T cells (Figure 3B). To evaluate whether the PSCA-CAR T cells can kill target cells *in vitro*, we used a luciferase-based killing assay where viable mel526(PSCA) and mel526(TARP) target cells express luciferase. Target cells were co-cultured with PSCA-CAR T cells or Mock T cells at different ratios for 2–4 days before evaluating the percentage of viable target cells by luminescence measurements. The PSCA-CAR T cells efficiently killed PSCA-expressing tumor cells from the three donors evaluated, but did not kill TARP-expressing tumor cells (Figure 3C). The Mock T cells did not exhibit any unspecific cytotoxic activity against the target cells when compared to PSCA-CAR T cells (Figure 3D). The experiments presented in Figure 3D were performed with PBMCs isolated from different donors when compared to the experiments with PSCA-CAR T cells against relevant or irrelevant targets (C).

### Systemic administration of PSCA-CAR T cells delay tumor growth and prolong survival of mice with subcutaneous PSCA-expressing tumors

**Figure 4:**
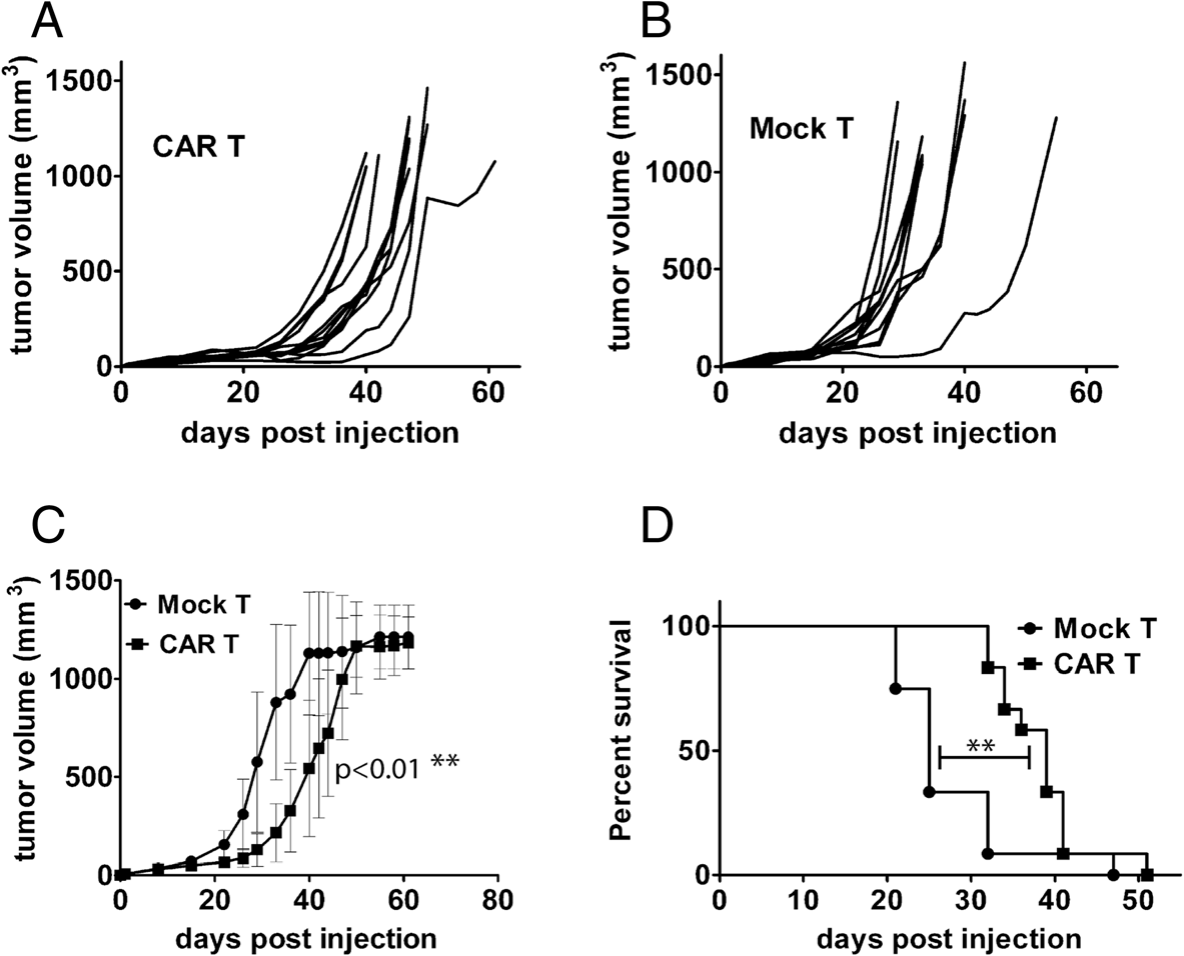
**Systemic treatment of mice with subcutaneous tumors with PSCA-CAR-engineered T cells leads to delayed tumor growth and prolonged survival.** Nude NMRI mice were injected subcutaneously with 3 × 10^6^ mel526(PSCA) cells. One, seven and fourteen days later the mice received intravenous injection of 1 × 10^7^ PSCA-CAR-transduced T cells (CAR T) or Mock-transduced T cells (Mock T). Twelve mice per group were used and tumor volumes were measured by caliper every second day. One experiment out of two is presented. **(A)** Tumor volume for individual mice treated with CAR T cells (n = 12). **(B)** Tumor volume for individual mice treated with Mock T cells (n = 12). **(C)** Pooled data on tumor volume for PSCA-CAR T cell-treated and Mock T cell-treated mice shows significant difference in tumor volume (p < 0.01, paired t-test). **(D)** Kaplan-Meier survival analysis shows significant difference in survival for mice treated with PSCA-CAR T cells and Mock T cells (p < 0.01, log rank test).

Next we wanted to evaluate the ability of the PSCA-CAR T cells to control tumor growth *in vivo*. Mel526(PSCA) tumor cells were implanted subcutaneously in *nude* mice and PSCA-CAR-transduced T cells or Mock lentivirus-transduced T cells were infused systemically three times, one week apart, by intravenous injections. The tumor sizes of twelve individual mice treated with PSCA-CAR T cells are shown in Figure 4A and the tumor sizes of twelve individual mice treated with Mock T cells are shown in Figure 4B. As an example, by day 38, two out of the twelve mice treated with PSCA-CAR T cells had to be sacrificed while at the same day eleven out of twelve mice treated by Mock T cells had already been sacrificed. The pooled data showed significantly smaller tumor volumes for mice treated with PSCA-CAR T cells compared to mice treated with Mock T cells (Figure 4C). Furthermore, the survival analysis showed significantly prolonged survival for mice treated with PSCA-CAR T cells compared to mice treated with Mock T cells (Figure 4D).

## Discussion

PSCA is a tissue-restricted antigen highly expressed on primary and metastatic prostate cancer cells *in vivo*[12,24]. It may therefore be an appropriate target for cancer immunotherapy [24]. Fully humanized antibodies against PSCA are now in clinical trial for prostate cancer but do not lead to cure [18]. The potential of therapeutic T cells to traffic to sites of disease, expand and persist remains a major advantage compared with antibodies. In fact, complete objective remissions have been observed for some cancer patients when autologous, engineered T cells have been used for treatment [3,10,11].

A number of CARs have recently been developed against PSCA [21,23,25,26]. Most publications on PSCA-CAR T cells use PSCA-transfected target cell lines to show T cell activity and only one publication show reactivity against a pancreatic tumor cell line with endogenous PSCA expression [23]. It should be noted that we did not detect PSCA expression on the surface of any prostate cancer nor pancreatic cancer cell line *in vitro*. Neither did we detect any PSCA expression on cultured primary prostate epithelial cells at different passages. Furthermore, we did not detect any PSCA expression on xenografted pancreatic cancer cell lines that were examined. Therefore, we were limited to use transduced target cells for PSCA-CAR T cell evaluation. We chose to use stable lentiviral transduction instead of transcient transfection, which can give unnaturally high levels of transgene expression.

There is only one report were PSCA-CAR-engineered T cells has been used in an *in vivo* model, in that case highly immunodeficient NSG mice with transplanted human tumors transduced to express PSCA [26]. Significant reduction in tumor growth rate was observed when the authors transferred T cells engineered with both a CAR that provides suboptimal activation upon binding of one antigen, PSCA, and a chimeric costimulatory receptor that recognizes a second antigen, PSMA, or vice versa. The authors further showed that co-transduced T cells destroy tumors that express both antigens but do not affect tumors expressing either antigen alone [26].

Herein, we used a third generation CAR against PSCA and we also show significant delay in tumor growth rate and significantly prolonged survival of *nude* mice. However, adoptive transfer of PSCA-CAR T cells alone did not cure any tumor-bearing mice. Whole-body irradiation as a preconditioning treatment before adoptive T cell transfer together with supportive administration of IL-2 have shown significantly improved results in mice [27]. It is therefore promising that our PSCA-CAR-engineered T cells are able to delay tumor growth *in vivo* without irradiation preconditioning or IL-2 support, though it may be beneficial to combine those treatments in the future for better effects. More experiments are needed to determine how long PSCA-CAR-engineered T cells persist or whether they proliferate at the tumor site. For example, T cells with longer telomeres that have high capacity to proliferate have been correlated with better prognosis for the patients receiving adoptive T cell transfer [28]. It may therefore be important to analyze the telomeres as well the phenotype of T cells and possibly select an optimal T cell subpopulation for genetic engineering and transfer. The method of T cell activation before transduction as well as the condition for *in vitro* culture of engineered T cells may also affect the performance of adoptively transferred CAR T cells.

## Conclusions

We confirm others finding that adoptive transfer of PSCA-CAR T cells is a potentially promising approach to treat prostate cancer. Although the expression of PSCA-CAR on the surface of the transduced T cells was intermediate high, almost all T cells were expressing the CAR, Figure 1B. Our experiments therefore indicate that even low level of expression of the CAR may be sufficient for T cell activation and T cell-mediated killing. Although in adoptive T cell transfer only highly reactive clones are selected (secreting more than 200 pg/ml IFN-γ after co-culture with targets), no correlation between IFN-γ secretion and persistence and efficacy of the cells *in vivo* has been found [29].

Prostate cancer has, like most cancers, an immunosuppressive tumor microenvironment [30] and it is important to have highly active T cells that will be able to proliferate and kill tumors also in this harsh environment. Therefore, our future focus will be on enhancing the resistance of the PSCA-CAR T cells to immunosuppressive factors.

## Abbreviations

CAR: Chimeric antigen receptor; PSCA: Prostate stem cell antigen; CD: Cluster of differentiation; IFN: Interferon; IgG (H + L): Immunoglobulin heavy and light chain; IL: Interleukin; TIL: Tumor infiltrating lymphocyte; TCR: T cell receptor; HLA: Human leukocyte antigen; ScFv: Single chain antibody fragment; CLL: Chronic lymphocytic leukemia; ALL: Acute lymphocytic leukemia; T2A: *Thosea asigna* virus 2A; Luc: *Firefly* luciferase; copGFP: *Copepod* green fluorescent protein; TARP: T cell receptor γ-chain alternate reading frame protein; PE: Phycoerythrin; APC: Allophycocyanin; FITC: Fluorescein isothiocyanate; SFFV: Spleen-focus forming virus; LAMP: Lysosomal-associated membrane protein.

## Competing interests

The authors declare that they have no competing interests.

## Authors’ contributions

VH, MR, JL and ME designed research; VH, MR and JL performed research and analyzed the data; VH, MR and ME wrote the paper. All authors read and approved the final manuscript.

## Authors’ information

Mohanraj Ramachandran and Justyna Leja shared authorship.

## Pre-publication history

The pre-publication history for this paper can be accessed here:

http://www.biomedcentral.com/1471-2407/14/30/prepub

## Supplementary Material

Additional file 1 Methods. Click here for file

Additional file 2: Figure S1 PSCA expression was not detected on various prostate and pancreatic cancer cells. A) PSCA expression was analyzed by flow cytometry on the surface of the human prostate cancer cell lines LNCaP, DU145, PC3, VCaP and on primary prostate epithelial cells of different passage (p). B) PSCA expression was analyzed by flow cytometry on the surface of human pancreatic cancer cell lines AsPc1, CaPan1, CaPan2 and Panc1 C) PSCA expression was analyzed by flow cytometry on the surface of the transduced mel526(PSCA) target cells and transduced mel526(TARP) control cells. Grey filled histograms represent anti-PSCA-stained cells while white filled histograms represent isotype control antibody staining. Click here for file

Additional file 3: Figure S2 PSCA expression was not detected on xenografted pancreatic cancer cells. PSCA expression was analyzed by flow cytometry on the surface of the pancreatic cell lines AsPc1 and CaPan2 after they have been grown subcutaneously in nude mice. Grey filled histograms represent anti-PSCA-stained cells while white filled histograms represent isotype control antibody staining. Click here for file

## References

[B1] Rosenberg SA Restifo NP Yang JC Morgan RA Dudley ME Adoptive cell transfer: a clinical path to effective cancer immunotherapy Nat Rev Cancer 2008 8 4 299 308 10.1038/nrc2355 18354418 PMC2553205

[B2] Essand M Loskog AS Genetically engineered T cells for the treatment of cancer J Intern Med 2013 273 2 166 181 10.1111/joim.12020 23198862 PMC3607417

[B3] Morgan RA Dudley ME Wunderlich JR Hughes MS Yang JC Sherry RM Royal RE Topalian SL Kammula US Restifo NP Zheng Z Nahvi A de Vries CR Rogers-Freezer LJ Mavroukakis SA Rosenberg SA Cancer regression in patients after transfer of genetically engineered lymphocytes Science 2006 314 5796 126 129 10.1126/science.1129003 16946036 PMC2267026

[B4] Sadelain M Brentjens R Riviere I The promise and potential pitfalls of chimeric antigen receptors Curr Opin Immunol 2009 21 2 215 223 10.1016/j.coi.2009.02.009 19327974 PMC5548385

[B5] Kowolik CM Topp MS Gonzalez S Pfeiffer T Olivares S Gonzalez N Smith DD Forman SJ Jensen MC Cooper LJN CD28 Costimulation provided through a CD19-specific chimeric antigen receptor enhances in vivo persistence and antitumor efficacy of adoptively transferred T cells Cancer Res 2006 66 22 10995 11004 10.1158/0008-5472.CAN-06-0160 17108138

[B6] Song D-G Ye Q Carpenito C Poussin M Wang L-P Ji C Figini M June CH Coukos G Powell DJ In vivo persistence, tumor localization, and antitumor activity of CAR-engineered T cells is enhanced by costimulatory signaling through CD137 (4-1BB) Cancer Res 2011 71 13 4617 4627 10.1158/0008-5472.CAN-11-0422 21546571 PMC4140173

[B7] Pule MA Straathof KC Dotti G Heslop HE Rooney CM Brenner MK A chimeric T cell antigen receptor that augments cytokine release and supports clonal expansion of primary human T cells Mol Ther 2005 12 5 933 941 10.1016/j.ymthe.2005.04.016 15979412

[B8] Song DG Ye Q Poussin M Harms GM Figini M Powell DJ Jr CD27 costimulation augments the survival and antitumor activity of redirected human T cells in vivo Blood 2012 119 3 696 706 10.1182/blood-2011-03-344275 22117050

[B9] Wang J Jensen M Lin Y Sui X Chen E Lindgren CG Till B Raubitschek A Forman SJ Qian X James S Greenberg P Riddell S Press OW Optimizing adoptive polyclonal T cell immunotherapy of lymphomas, using a chimeric T cell receptor possessing CD28 and CD137 costimulatory domains Hum Gene Ther 2007 18 8 712 725 10.1089/hum.2007.028 17685852

[B10] Kalos M Levine BL Porter DL Katz S Grupp SA Bagg A June CH T cells with chimeric antigen receptors have potent antitumor effects and can establish memory in patients with advanced leukemia Sci Transl Med 2011 3 95 95ra73 21832238 10.1126/scitranslmed.3002842 PMC3393096

[B11] Grupp SA Kalos M Barrett D Aplenc R Porter DL Rheingold SR Teachey DT Chew A Hauck B Wright JF Milone MC Levine BL June CH Chimeric antigen receptor-modified T cells for acute lymphoid leukemia The New England J of Med 2013 368 16 1509 1518 10.1056/NEJMoa1215134 PMC4058440 23527958

[B12] Gu Z Thomas G Yamashiro J Shintaku IP Dorey F Raitano A Witte ON Said JW Loda M Reiter RE Prostate stem cell antigen (PSCA) expression increases with high gleason score, advanced stage and bone metastasis in prostate cancer Oncogene 2000 19 10 1288 1296 10.1038/sj.onc.1203426 10713670

[B13] Zhang KQ Yang F Ye J Jiang M Liu Y Jin FS Wu YZ A novel DNA/peptide combined vaccine induces PSCA-specific cytotoxic T-lymphocyte responses and suppresses tumor growth in experimental prostate cancer Urology 2012 79 6 1410 e1417-1413 22513035 10.1016/j.urology.2012.02.011

[B14] Kiessling A Schmitz M Stevanovic S Weigle B Holig K Fussel M Fussel S Meye A Wirth MP Rieber EP Prostate stem cell antigen: identification of immunogenic peptides and assessment of reactive CD8+ T cells in prostate cancer patients Int J Cancer 2002 102 4 390 397 10.1002/ijc.10713 12402309

[B15] Forsberg O Carlsson B Malmstrom PU Ullenhag G Totterman TH Essand M High frequency of prostate antigen-directed T cells in cancer patients compared to healthy age-matched individuals Prostate 2009 69 1 70 81 10.1002/pros.20858 18814178

[B16] Leyton JV Olafsen T Lepin EJ Hahm S Bauer KB Reiter RE Wu AM Humanized radioiodinated minibody for imaging of prostate stem cell antigen–expressing tumors Clin Cancer Res 2008 14 22 7488 7496 10.1158/1078-0432.CCR-07-5093 19010866 PMC2720761

[B17] Antonarakis E Carducci M Eisenberger M Denmeade S Slovin S Jelaca-Maxwell K Vincent M Scher H Morris M Phase I rapid dose-escalation study of AGS-1C4D4, a human anti-PSCA (prostate stem cell antigen) monoclonal antibody, in patients with castration-resistant prostate cancer: a PCCTC trial Cancer Chemother Pharmacol 2012 69 3 763 771 10.1007/s00280-011-1759-9 22020316 PMC3586214

[B18] Morris MJ Eisenberger MA Pili R Denmeade SR Rathkopf D Slovin SF Farrelly J Chudow JJ Vincent M Scher HI Carducci MA A phase I/IIA study of AGS-PSCA for castration-resistant prostate cancer Ann Oncol 2012 23 10 2714 2719 10.1093/annonc/mds078 22553195 PMC3457748

[B19] Olafsen T Gu Z Sherman MA Leyton JV Witkosky ME Shively JE Raubitschek AA Morrison SL Wu AM Reiter RE Targeting, imaging, and therapy using a humanized antiprostate stem cell antigen (PSCA) antibody J Immunother 2007 30 4 396 405 10.1097/CJI.0b013e318031b53b 17457214

[B20] Yvon E Del Vecchio M Savoldo B Hoyos V Dutour A Anichini A Dotti G Brenner MK Immunotherapy of metastatic melanoma using genetically engineered GD2-specific T cells Clin Cancer Res 2009 15 18 5852 5860 10.1158/1078-0432.CCR-08-3163 19737958 PMC2745508

[B21] Zheng Z Chinnasamy N Morgan RA Protein L: a novel reagent for the detection of chimeric antigen receptor (CAR) expression by flow cytometry J Transl Med 2012 10 29 10.1186/1479-5876-10-29 22330761 PMC3299624

[B22] Taylor RM Severns V Brown DC Bisoffi M Sillerud LO Prostate cancer targeting motifs: expression of alphanu beta3, neurotensin receptor 1, prostate specific membrane antigen, and prostate stem cell antigen in human prostate cancer cell lines and xenografts Prostate 2012 72 5 523 532 10.1002/pros.21454 21748756 PMC4366051

[B23] Katari UL Keirnan JM Worth AC Hodges SE Leen AM Fisher WE Vera JF Engineered T cells for pancreatic cancer treatment HPB: the Off J of the Int Hepato Pancreato Biliary Assoc 2011 13 9 643 650 10.1111/j.1477-2574.2011.00344.x PMC3183449 21843265

[B24] Jalkut MW Reiter RE Role of prostate stem cell antigen in prostate cancer research Curr Opin Urol 2002 12 5 401 406 10.1097/00042307-200209000-00006 12172427

[B25] Morgenroth A Cartellieri M Schmitz M Gunes S Weigle B Bachmann M Abken H Rieber EP Temme A Targeting of tumor cells expressing the prostate stem cell antigen (PSCA) using genetically engineered T-cells Prostate 2007 67 10 1121 1131 10.1002/pros.20608 17492652

[B26] Kloss CC Condomines M Cartellieri M Bachmann M Sadelain M Combinatorial antigen recognition with balanced signaling promotes selective tumor eradication by engineered T cells Nat Biotechnol 2013 31 1 71 75 23242161 10.1038/nbt.2459 PMC5505184

[B27] Kochenderfer JN Yu ZY Frasheri D Restifo NP Rosenberg SA Adoptive transfer of syngeneic T cells transduced with a chimeric antigen receptor that recognizes murine CD19 can eradicate lymphoma and normal B cells Blood 2010 116 19 3875 3886 10.1182/blood-2010-01-265041 20631379 PMC2981541

[B28] Rosenberg SA Yang JC Sherry RM Kammula US Hughes MS Phan GQ Citrin DE Restifo NP Robbins PF Wunderlich JR Morton KE Laurencot CM Steinberg SM White DE Dudley ME Durable complete responses in heavily pretreated patients with metastatic melanoma using T-cell transfer immunotherapy Clin Cancer Res 2011 17 13 4550 4557 10.1158/1078-0432.CCR-11-0116 21498393 PMC3131487

[B29] Besser MJ Shapira-Frommer R Treves AJ Zippel D Itzhaki O Schallmach E Kubi A Shalmon B Hardan I Catane R Segal E Markel G Apter S Nun AB Kuchuk I Shimoni A Nagler A Schachter J Minimally cultured or selected autologous tumor-infiltrating lymphocytes after a lympho-depleting chemotherapy regimen in metastatic melanoma patients J Immunother 2009 32 4 415 423 10.1097/CJI.0b013e31819c8bda 19342963

[B30] Barach YS Lee JS Zang XX T cell coinhibition in prostate cancer: new immune evasion pathways and emerging therapeutics Trends Mol Med 2011 17 1 47 55 10.1016/j.molmed.2010.09.006 PMC3039708 20971039

