# Supplementary material for: Systemic treatment with CAR-engineered T cells against PSCA delays subcutaneous tumor growth and prolongs survival of mice
Source: BMC Cancer. 2014 Jan 18;14:30. doi: 10.1186/1471-2407-14-30 (PMC3899402; doi:10.1186/1471-2407-14-30)
Supplement: Additional file 1 — Methods. [file 1471-2407-14-30-S1.docx]

**Supplementary information**

**Methods**

**Target cell lines**

Primary prostate epithelial cells (HPEpiC) (3H Biomedical AB, Uppsala, Sweden) were grown in prostate epithelial cell medium (3H Biomedical AB). The prostate cancer cell lines LNCaP, DU145, PC3 and VCaP were grown in RPMI-1640 supplemented with 10% FBS, 10 mM HEPES, 1 mM sodium pyruvate and 0.5% penicillin/streptomycin (PEST) (Invitrogen). The pancreatic cancer cell lines CaPan1 and CaPan2 were grown in RPMI 1640 supplemented with 2 mM L-glutamine, 10 mM HEPES and 10% FBS. The pancreatic cancer cell line AsPc1 was grown in RPMI-1640 supplemented with 2 mM L-glutamine, 10 mM HEPES, and 20% FBS. The pancreatic cancer cell line Panc1 was grown in DMEM supplemented with 10% FBS.

**Subcutaneous pancreatic tumors**

Nude NMRI mice (Taconic, Denmark) were injected subcutaneously (hind flank) with 5x10^6^ human pancreatic cancer cells, AsPc1 or CaPan2. Animals were sacrificed 21 days after tumor inoculation, when tumors reached approximately 150-200 mm^3^. Tumor samples were collected, transferred to a sterile Petri-dish, cut into small pieces with a scalpel and placed in digestion buffer (Ham’s F-10, Collagenase (2 mg/ml), DNase I (0.05 mg/ml), 1.5% BSA, 1.25 mM CaCl_2_] for 60 min at 37°C, while shaking. The digested tumor specimens were filtered through a sterile cell strainer (BD Bioscience) with Versene solution (1xPBS buffer with 0.53 mM EDTA), collected and centrifuged (1500 rpm, 5 min, RT). The supernatant was removed and the single cells were resuspended in Versene solution.

**Flow cytometry of target cells for PSCA expression**

All the target cells mentioned above were incubated with Mouse Fc Block (BD Bioscience) for 5 min at 4°C stained with a mouse monoclonal PE-conjugated anti-human PSCA antibody (Santa Cruz) for 30 min at 4°C followed by flow cytometry analysis. Mel526(PSCA) and mel526(TARP) cells, described in the main text, were stained with a mouse monoclonal anti-human PSCA antibody (Santa Cruz), followed by staining with APC-conjugated goat anti-mouse IgG antibody. Unspecific binding was measured by matched isotype PE or APC-labeled antibodies (BD Biosciences).

**Figure Legends**

**Supplementary Figure S1. PSCA expression was not detected on various prostate and pancreatic cancer cells. A)** PSCA expression was analyzed by flow cytometry on the surface of the human prostate cancer cell lines LNCaP, DU145, PC3, VCaP and on primary prostate epithelial cells of different passage. **B)** PSCA expression was analyzed by flow cytometry on the surface of the human pancreatic cancer cell lines AsPc1, CaPan1, CaPan2 and Panc1 **C)** PSCA expression was analyzed by flow cytometry on the surface of the transduced mel526(PSCA) target cells and transduced mel526(TARP) control cells. Grey filled histograms represent anti-PSCA-stained cells while white filled histograms with black lines represent isotype control antibody staining.

**Supplementary Figure S2. PSCA expression was not detected on xenografted pancreatic cancer cells.** PSCA expression was analyzed by flow cytometry on the surface of the pancreatic cell lines AsPc1 and CaPan2 after they have been grown subcutaneously in nude mice. Grey filled histograms represent anti-PSCA-stained cells while white filled histograms represent isotype control antibody staining.
