# Supplementary figures and images for: Systemic treatment with CAR-engineered T cells against PSCA delays subcutaneous tumor growth and prolongs survival of mice
Source: BMC Cancer. 2014 Jan 18;14:30. doi: 10.1186/1471-2407-14-30 (PMC3899402; doi:10.1186/1471-2407-14-30)

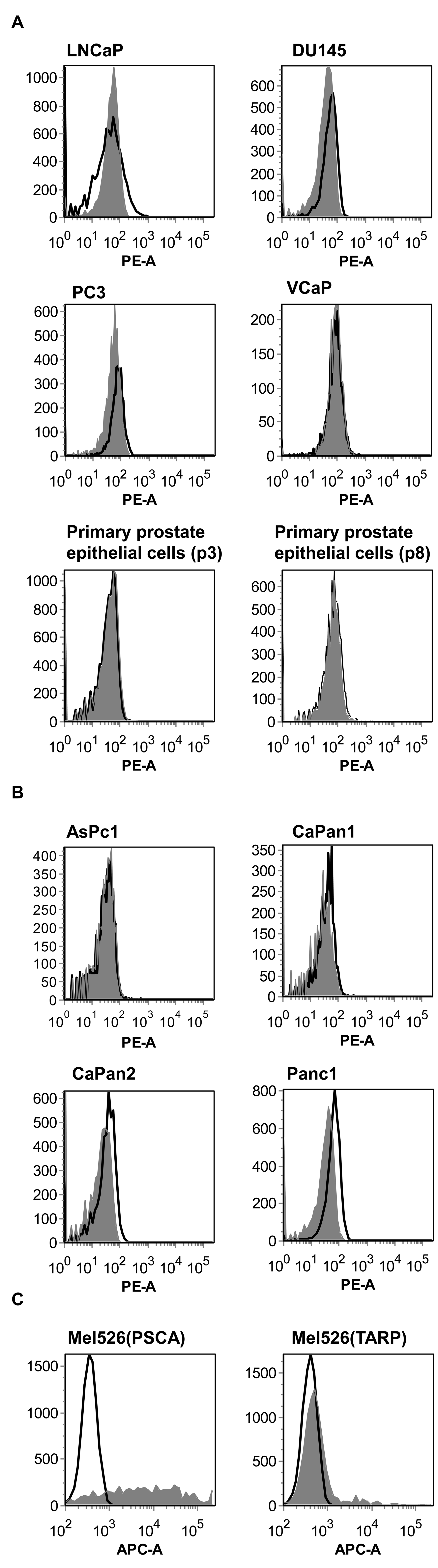

Supplement: Additional file 2: Figure S1 — PSCA expression was not detected on various prostate and pancreatic cancer cells. A) PSCA expression was analyzed by flow cytometry on the surface of the human prostate cancer cell lines LNCaP, DU145, PC3, VCaP and on primary prostate epithelial cells of different passage (p). B) PSCA expression was analyzed by flow cytometry on the surface of human pancreatic cancer cell lines AsPc1, CaPan1, CaPan2 and Panc1 C) PSCA expression was analyzed by flow cytometry on the surface of the transduced mel526(PSCA) target cells and transduced mel526(TARP) control cells. Grey filled histograms represent anti-PSCA-stained cells while white filled histograms represent isotype control antibody staining. [file 1471-2407-14-30-S2.tiff]

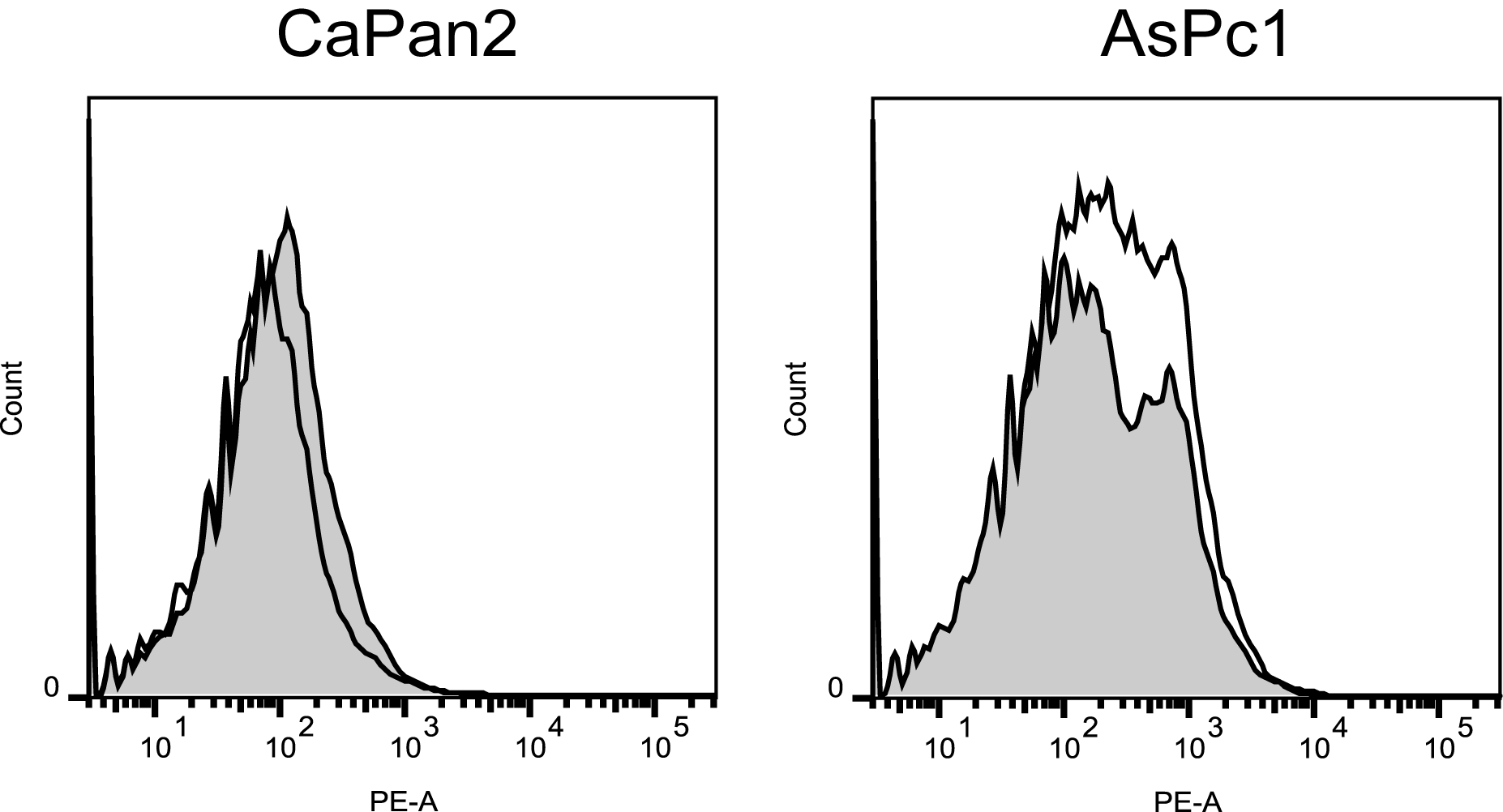

Supplement: Additional file 3: Figure S2 — PSCA expression was not detected on xenografted pancreatic cancer cells. PSCA expression was analyzed by flow cytometry on the surface of the pancreatic cell lines AsPc1 and CaPan2 after they have been grown subcutaneously in nude mice. Grey filled histograms represent anti-PSCA-stained cells while white filled histograms represent isotype control antibody staining. [file 1471-2407-14-30-S3.tiff]
